# A Comprehensive Epidemiological Research for Clinical *Vibrio parahaemolyticus* in Shanghai

**DOI:** 10.3389/fmicb.2017.01043

**Published:** 2017-06-08

**Authors:** Huan Li, Rong Tang, Yang Lou, Zelin Cui, Wenjing Chen, Qing Hong, Zhaohuan Zhang, Pradeep K. Malakar, Yingjie Pan, Yong Zhao

**Affiliations:** ^1^College of Food Science and Technology, Shanghai Ocean UniversityShanghai, China; ^2^Shanghai General HospitalShanghai, China; ^3^Laboratory of Quality and Safety Risk Assessment for Aquatic Products on Storage and Preservation (Shanghai), Ministry of AgricultureShanghai, China; ^4^Shanghai Engineering Research Center of Aquatic-Product Processing and PreservationShanghai, China

**Keywords:** *Vibrio parahaemolyticus*, diarrhoea samples, virulence, genetic diversity, antimicrobial susceptibility

## Abstract

*Vibrio parahaemolyticus* is one of the most important pathogen for seafood-borne gastroenteritis in Shanghai and the rest of the world. A total of 42 *V. parahaemolyticus* strains were isolated from 1900 fecal specimens collected from patients in Shanghai hospital presenting from January 2014 to December 2015. All isolates were evaluated for potential virulence factors [*tdh*, *trh*, and type three secretion system (T3SS) genes], typed using multilocus sequence typing (MLST) and screened for antimicrobial resistance phenotype and genotype. And for the first time, the relationship between virulence, genetic diversity and antimicrobial resistance of these isolates were identified. The results showed that 37 isolates carried the *tdh* gene (88.1%) and only seven isolates were positive for the *trh* gene. The T3SS1 and T3SS2 genes were detected in all strains and only *trh*-positive isolates are also containing the T3SS2β genes. MLST analysis of the 42 Shanghai isolates identified 20 sequence types (STs) with 16 novel STs and that these clinical *V. parahaemolyticus* strains showed high degrees of genetic diversity. All isolates expressed high levels of resistance against Ampicillin (100.0%), Streptomycin (100.0%), Cephazolin (92.9%), Kanamycin (92.8%) and Amikacin (90.5%), and eight out of 38 resistance genes (*SHV*, *tet*(B), *str*A, *qnr*A, *gry*A, *qnr*B, *sul*I, *sul*II) were detected in at least two isolates. This study confirms that antimicrobial resistance of clinical *V. parahaemolyticus* isolates is greater than those of environmental isolates. Furthermore, no clear correlation between antimicrobial resistance and virulence or genetic diversity was found in this study. These results add to epidemiological data of clinical *V. parahaemolyticus* isolates in Shanghai and highlight the need for additional mechanistic studies, especially antimicrobial resistance, to reduce the burden of disease caused by this pathogen in China.

## Introduction

Illnesses caused by foodborne pathogens are an increasingly critical public health concern (Velusamy et al., 2010). *Vibrio parahaemolyticus* is recognized as a major foodborne pathogen for causing gastroenteritis worldwide, especially in coastal countries and regions (McLaughlin et al., 2005; Su and Liu, 2007; Letchumanan et al., 2014; Hubbard et al., 2016). Clinical symptom of *V. parahaemolyticus* infections include diarrhea, abdominal cramps, vomiting and fever, which also progresses to septicemia can sometimes lead to death in patients (Scallan et al., 2011; Lopatek et al., 2015). In 1950, this pathogen was first discovered in Japan, which resulted 272 illnesses with 20 deaths (Fujino et al., 1953). From 1997 to 2000, 84 food poisoning outbreaks caused by *V. parahaemolyticus* were recorded in Spain (Fournier and Ogata, 2005). From 2000 to 2008, it was reported around 35,000 *V. parahaemolyticus* infections annually in US (Haendiges et al., 2015). In China, during 2003 to 2008, this microorganism has caused 9041 illnesses and 3948 hospitalizations (Wu et al., 2014). It is essential to gather the epidemiological data of *V. parahaemolyticus* to reduce the burden of disease caused by this pathogen in China.

Virulence of *V. parahaemolyticus* is primarily attributed to the production of a thermostable direct haemolysin (TDH), TDH-related haemolysin (TRH) and two type III secretion systems, T3SS (Makino et al., 2003; Ritchie et al., 2012; Zhang et al., 2013). TDH and TRH are encoded by the *tdh* gene and *trh* gene, respectively (Letchumanan et al., 2014). Although the specific actions of these genes in human infection remain unknown, the relevance between pathogenicity of *V. parahaemolyticus* and the presence of *tdh* and *trh* is well recognized (Broberg et al., 2011; Ceccarelli et al., 2013). Contamination of foods with *tdh*- and/or *trh*-positive *V. parahaemolyticus* strains is considered a public health risk (Pazhani et al., 2014). The two T3SS systems in *V. parahaemolyticus* are known as T3SS1 and T3SS2 (Wang et al., 2015). T3SS1 is encoded by the first pathogenicity island on chromosome I and is involved in cytotoxicity (Paranjpye et al., 2012). T3SS2 is located on chromosome II and is also encoded by a pathogenicity island. As a newly identified type of secretion system, T3SS2 appears to be associated with enterotoxicity and cytotoxicity, in experiments conducted in vitro and in intestinal cell lines (Ritchie et al., 2012). Currently, it is possible to detect the presence of *tdh*, *trh* and T3SS genes in *V. parahaemolyticus* isolates by PCR-based methods (West et al., 2013).

*Vibrio parahaemolyticus* strains exhibit high genetic diversity due to high rates of recombination and mutation, which caused potential infection risk for human health (Ludeke et al., 2015). A number of molecular typing methods have been used to determine the molecular epidemiology of *V. parahaemolyticus*, and these methods include multilocus sequence typing (MLST), serotyping, and pulsed-field gel electrophoresis (PFGE) (Banerjee et al., 2014; Xu et al., 2015). MLST has proven to be powerful tool for investigating the prevalence and diversity of *V. parahaemolyticus* strains in recent years (Wu et al., 2016). MLST is based on the sequencing of seven housekeeping genes and can be analyzed directly via the internet (Gonzalez-Escalona et al., 2008). MLST is commonly used for identifying the relationship between isolates in public database and has proven to be an important method for investigation of the evolution and epidemiology of *V. parahaemolyticus* (Banerjee et al., 2014).

Antimicrobials are used in the treatment of infectious diseases and improper or enhanced application of antimicrobials leads to development of antimicrobial resistant (AMR) bacteria (Yano et al., 2014). The Economic Forum for Global Risks indicates that the problem of AMR is projected to be one of the greatest threats to human health in the future (Koser et al., 2014; Blair et al., 2015). The critical factors for the emergence of AMR are antimicrobial resistance genes (ARGs) which can be transferred by the horizontal gene transfer (Thomas and Nielsen, 2005). ARGs are emerging contaminants posing a potential worldwide human health risk (Allen et al., 2010; Lou et al., 2016). It is vital to monitor the AMR and ARGs of *V. parahaemolyticus* strains, which can be used for disease management and reducing the burden of disease caused by this pathogen.

Shanghai is one of the largest prosperous cities in China with high annual consumption of seafood and many cases of *V. parahaemolyticus* infections (Zhang and Orth, 2013; Qi et al., 2016), which have become a potential threat for human health. The researches for *V. parahaemolyticus* strains isolated from aquatic products are widely reported (Wang et al., 2011; Guo et al., 2013; He et al., 2015, 2016; Lou et al., 2016; Hu and Chen, 2016; Yu et al., 2016; Zhang et al., 2017), while studies on clinical isolates has been poorly documented (Zhang and Orth, 2013; Qi et al., 2016).

The main objectives of this study are to monitor the virulence, genetic diversity and antimicrobial susceptibility of clinical *V. parahaemolyticus* isolates from Shanghai. We hope to provide reliable information, for assessing the genetic traits and the antimicrobial resistance risk of *V. parahaemolyticus* strains, and for better management of foodborne infections in Shanghai.

## Materials and Methods

### Specimen Collection and Bacteria Isolation

A total of 1900 fecal specimens were collected by Shanghai hospital from patients who presented with acute diarrhea to gastroenteritis outpatient clinics during the period from January 2014 through to December 2015. These fecal samples were placed in sterile sealed plastic bags and stored at 4°C prior to further analysis. Confirmation of *V. parahaemolyticus* samples were performed using standard culture methods (ISO, 2007).

Briefly, 25 g of each fecal specimen was homogenized for 2 min in a stomacher 400 with 225 mL of alkaline peptone water (APW; Beijing Land Bridge Technology Company Ltd., Beijing, China) containing 3% NaCl, and incubated at 37°C for 16-18 h. After incubation, a loop from the top 1 cm was streaked onto thiosulfate-citrate-bile salts-sucrose (TCBS; Beijing Land Bridge Technology Company Ltd., Beijing, China) agar plates and incubated at 37°C for 18–24 h. Presumptive individual bacterial colony (green or blue green colony, 2–3 mm in diameter) were grown in 10 ml tryptic soy broth (TSB; Beijing Land Bridge Technology Company Ltd., Beijing, China) supplemented with 3.0% NaCl and incubated at 37°C for 18–24 h. After cultivation, the bacterial liquid and 50% glycerol in the proportion of 1:1 were placed in a glycerol tube and stored at –80°C for further analysis.

### DNA Extraction

DNA extraction of all presumptive *V. parahaemolyticus* isolates was performed using the TIANamp Bacteria DNA Kit (Tiangen Biotech Beijing Co., Ltd, Beijing, China), in accordance to the manufacturer’s recommended protocols and then stored it at –20°C prior to PCR analysis.

### Identification of *Vibrio parahaemolyticus*

The presumptive *V. parahaemolyticus* isolates were tested for the presence of the species specific gene *tlh* by using polymerase chain reaction (PCR). Detection of *tlh* gene was carried out using the primer *tlh*-F (5- AAA GCG GAT TAT GCA GAA GCA CTG -3) and *tlh*-R (5- GCT ACT TTC TAG CAT TTT CTC TGC -3) as specified in (Food and Drug Administration [FDA], 2004). The reaction mixture for this PCR assay was performed in 25 μL, containing 1 μL of DNA template, 12.5 μL of PCR Mix (Sangon Biotech, Shanghai, China), 9.5 μL of dd H_2_O and 1 μL of each primer. The thermal-cycling program is as follows: initial denaturation at 94°C for 3 min, 25 cycles of 94°C for 1 min, 60°C for 1 min and 72°C for 2 min, and a final extension at 72°C for 3 min. Finally, PCR products were analyzed by agarose gel electrophoresis.

We also chose the API 20E system (BioMerieux, Inc., Durham, NC, United States) and DBI-08 (Beijing Land Bridge Technology Company Ltd., Beijing, China) to analyze and identify the *V. parahaemolyticus* isolates, according to the procedure described by the manufacturer and using *V. parahaemolyticus* ATCC 33847 as the reference strain (Croci et al., 2007; Li et al., 2016; Xie et al., 2016).

### Detection of Virulence-Associated Genes

Detection of the *V. parahaemolyticus* virulence genes *tdh* (West et al., 2013) and *trh* (Nilsson and Turner, 2016) were also performed by PCR. We designed a primer for detection of the *ureR* gene to study the variation of the *trh* gene as outlined in Nilsson and Turner, 2016. The *ureR* gene encodes for the transcriptional activator of the urease gene cluster located immediately upstream from *trh* and is widely reported to be genetically linked to *trh* (Nilsson and Turner, 2016). *V. parahaemolyticus* virulence associated genes of type III secretion system-1 (T3SS1) genes (VP1670 [*vscP*], VP1686 [*putative*], VP1689 [*vscK*] and VP1694 [*vscF*]), T3SS2α genes (VP1362 [*vopB2*], VP1339 [*vscC2*], VP1335 [*vscS2*] and VP1327 [*vopT*]) and the T3SS2β genes (*vscC2*, *vopB2*, *vopC*, *vscS2*) were tested by conventional PCR (Jones et al., 2012). In our study, the oligonucleotide primers were synthesized by Sangon Biotech (Sangon Biotech, Shanghai, China). Particularly worth mentioning is that the *V. parahaemolyticus* ATCC17802 (*trh*+) and ATCC33847(*tdh*+) were used as the reference strains, and distilled water was used as the negative control.

### Multilocus Sequence Typing

Seven housekeeping genes, *dnaE*, *gyrB*, *recA*, *dtdS*, *pntA*, *pyrC*, and *tnaA* (Supplementary Table S1), were used for *V. parahaemolyticus* characterization under the MLST scheme, PCR fragments were sequenced by Sangon Biotech (Sangon Biotech, Shanghai, China) and alignments of these sequences were determined using DNAMAN. The sequences were analyzed online^1^ to assign allele numbers and define sequence types (STs). New sequences for alleles and new ST profiles were submitted to the *V. parahaemolyticus* MLST database. Based on the relatedness of the STs, all of the isolates were subdivided into clonal complexes (CCs) or groups by eBURST program. Nucleotide sequence analyses were evaluated by MEGA5.1 program. In this study, the primary founder of a CC, a single locus variants (SLVs), double locus variants (DLVs), and singletons were defined as described previously (Han et al., 2015).

### Antimicrobial Susceptibility Testing

The antibiotic susceptibilities of the 42 isolates were assessed using the disk diffusion method on Mueller Hinton agar (MHA) (OXOID Limited, China) according to the guidelines of the Clinical and Laboratory Standards Institute (CLSI, 2015; Lou et al., 2016). Briefly, Muller–Hinton agar and a panel of 18 antibiotics disks were selected for resistance tests. The 18 common antimicrobials belonging to 6 classes used in this study were: β-lactam (ampicillin: AMP, amoxicillin-clavulanic: AMC, piperacillin: PRL, cefotaxime: CTX, ceftazidime: CAZ, cefoxitin: FOX, cephazolin: KZ, imipenem: IPM, meropenem: MEM), aminoglycoside (amikacin: AK, gentamicin: CN, kanamycin: K, streptomycin: S), tetracycline (tetracycline: TET), quinolone (ciprofloxacin: CIP, levofloxacin: LEV), sulfonamides (trimethoprim-sulfamethoxazole: SXT), chloramphenicol (chloramphenicol: C). The results were expressed as sensitive (S), intermediate (I), or resistant (R) according to the methods of the CLSI (CLSI, 2015). *Escherichia coli* ATCC 25922 was used as the quality control organism for the antimicrobial susceptibility testing.

### Evaluation of Antibiotic Resistance-Encoding Genes

The 38 antibiotic resistance genes (Supplementary Table S2) of six classes of antibiotics were identified by PCR, as previously described (Lou et al., 2016). All obtained PCR products were purified and sequenced by Sangon Biotech (Sangon Biotech, Shanghai, China). The acquired sequences were aligned and analyzed with the BLAST program^2^.

## Results

### Prevalence of *V. parahaemolyticus*

A total of 42 presumptive *V. parahaemolyticus* strains were isolated from 1900 fecal specimens (2.2%) collected from patients presenting in Shanghai hospital during January, 2014 to December, 2015. The PCR results showed all isolates were positive for the presence of the *tlh* gene (**Table 1**), which indicated that these 42 isolates were *V. parahaemolyticus* strains. And the results of API 20E and DBI-08 provided further evidence to the veracity of PCR outcomes, with all 42 isolates being identified with 99% confidence as *V. parahaemolyticus*. The demographic characteristics of patients about 42 *V. parahaemolyticus* isolates are also presented in **Table 1**. The 42 individuals enrolled in this research included 20 females and 22 males. The male patients’ ages ranged from 13 to 57, while for the female patients, the age distribution was more uniform in women whose median ages above 30 years-old.

**Table 1:** Information of 42 *Vibrio parahaemolyticus* clinical isolates.

Name	Gender	Age	*tlh*	*tdh*	*trh*	*ureR*
VPC1	Female	57	_+_	_+_	**–**	**–**
VPC2	Female	53	_+_	_+_	**–**	**–**
VPC15	Female	30	_+_	_+_	**–**	**–**
VPC16	Female	31	_+_	_+_	**–**	**–**
VPC17	Male	56	_+_	_+_	**–**	**–**
VPC18	Female	29	_+_	**–**	_+_	_+_
VPC19	Male	16	_+_	_+_	**–**	**–**
VPC20	Male	13	_+_	_+_	**–**	**–**
VPC21	Female	54	_+_	_+_	**–**	**–**
VPC22	Male	17	_+_	_+_	**–**	**–**
VPC25	Female	35	_+_	_+_	**–**	**–**
VPC26	Female	36	_+_	_+_	**–**	**–**
VPC27	Male	22	_+_	_+_	**–**	**–**
VPC28	Male	48	_+_	_+_	**–**	**–**
VPC29	Male	26	_+_	_+_	**–**	**–**
VPC32	Male	57	_+_	_+_	**–**	**–**
VPC33	Male	38	_+_	_+_	**–**	**–**
VPC34	Male	37	_+_	_+_	**–**	**–**
VPC35	Male	56	_+_	_+_	**–**	**–**
VPC36	Female	47	_+_	**–**	_+_	_+_
VPC37	Male	63	_+_	_+_	**–**	**–**
VPC38	Female	56	_+_	_+_	**–**	**–**
VPC40	Female	58	_+_	_+_	**–**	**–**
VPC41	Female	79	_+_	_+_	**–**	**–**
VPC42	Male	55	_+_	_+_	**–**	**–**
VPC43	Male	56	_+_	_+_	_+_	_+_
VPC44	Male	33	_+_	**–**	**–**	**–**
VPC45	Female	54	_+_	_+_	**–**	**–**
VPC46	Male	26	_+_	_+_	**–**	**–**
VPC47	Female	86	_+_	_+_	**–**	**–**
VPC48	Female	45	_+_	_+_	**–**	**–**
VPC49	Female	56	_+_	_+_	_+_	_+_
VPC50	Male	46	_+_	_+_	**–**	**–**
VPC51	Male	24	_+_	_+_	**–**	**–**
VPC54	Female	39	_+_	_+_	_+_	_+_
VPC55	Male	22	_+_	_+_	**–**	**–**
VPC85	Female	58	_+_	**–**	_+_	_+_
VPC89	Female	46	_+_	_+_	**–**	**–**
VPC90	Male	37	_+_	_+_	**–**	**–**
VPC94	Male	28	_+_	**–**	_+_	_+_
VPC97	Female	26	_+_	_+_	**–**	**–**
VPC100	Male	56	_+_	_+_	**–**	**–**


### Distribution of Virulence-Associated Genes

From our study, the hemolysin gene *tdh* was detected in most of the isolates (88.1%, 37/42), whereas the *trh* gene was present in only 7 strains (16.7%, 7/42). We further determine the distribution of the *trh* gene by the *ureR* gene. The *ureR* gene and the variable *trh* gene were observed in the same seven *V. parahaemolyticus* isolates. Of these, 3 of 42 (7.1%) clinical isolates were positive for both *tdh* and *trh*. Only one strains (2.4%, 1/42) from the diarrheal patients contained neither the *tdh* nor the *trh* gene.

The two types of T3SS complexes are features in the virulence mechanism of *V. parahaemolyticus* and the distribution of T3SS genes is presented in **Table 2**. T3SS1 genes were identified in all of the *V. parahaemolyticus* isolates and these samples contained all four T3SS1 genes (100%). All thirty-four of the *tdh*+/*trh*- and one of the *tdh*-/*trh*- clinical isolates contained all four genes of the T3SS2α genes. However, the isolates of *tdh*+/*trh*+ and *tdh*-/*trh*+ the detected percentage of all T3SS2α genes was only 33.3% and 50.0%, respectively. Additionally, four of the *tdh*-/*trh*+ isolates were amplified all four genes of the T3SS2β genes, followed by the isolates of *tdh*+/*trh*+ the detected percentage of all T3SS2β genes was 66.7%. Only one remaining *tdh*+/*trh*- isolate (VPC89) amplified all four T3SS2β genes but *vscC2*. As expected, one of the *tdh*-/*trh*- clinical isolate was evaluated in negative of all four T3SS2β genes. Overall, the T3SS2α-associated genes were most prevalent in *tdh*+ isolates (93.5%, 35/37), and the T3SS2β genes were detected prevalent in the *trh*+ clinical isolates (85.7%, 6/7).

**Table 2:** Distribution of T3SS genes among 42 clinical *V. parahaemolyticus* isolates.

Gene	No. of strains (n = 42)			
	
	*tdh* + *trh* – (n = 34)	*tdh* + *trh* + (n = 3)	*tdh* – *trh* + (n = 4)	*tdh* – *trh* – (n = 1)
**T3SS1**				
VP1670 (*vscP*)	34	3	4	1
VP1686 (*putative*)	34	3	4	1
VP1689 (*vscK*)	34	3	4	1
VP1694 (*vscF*)	34	3	4	1
All 4 genes present	34	3	4	1
**T3SS2α**				
VP1362 (*vopB2*)	34	2	3	1
VP1339 (*vscC2*)	34	1	3	1
VP1335 (*vscS2*)	34	1	2	1
VP1327 (*vopT*)	34	1	2	1
All 4 genes present	34	1	2	1
**T3SS2β**				
*vscC2*	0	3	4	0
*vopB2*	1	3	4	0
*vopC*	1	2	4	0
*vscS2*	1	3	4	0
All 4 genes present	0	2	4	0


### Multilocus Sequence Typing Analysis

**FIGURE 1:**
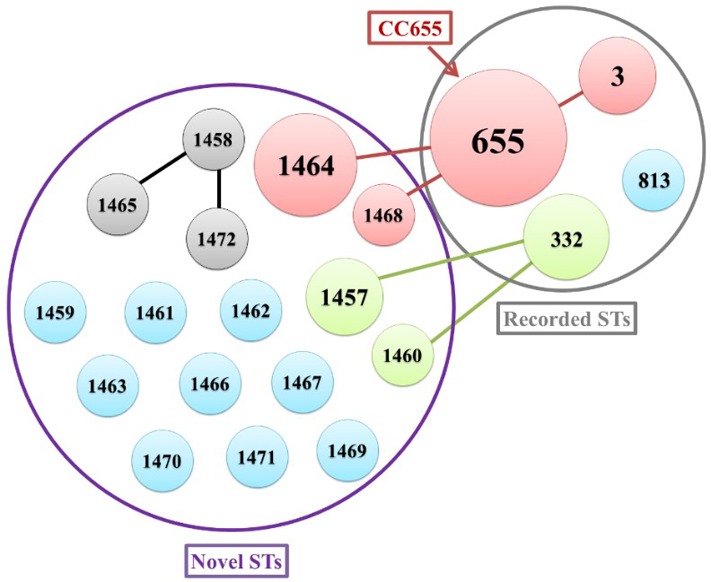
Multilocus sequence typing (MLST) of 42 *V. parahaemolyticus* clinical isolates.

**FIGURE 2:**
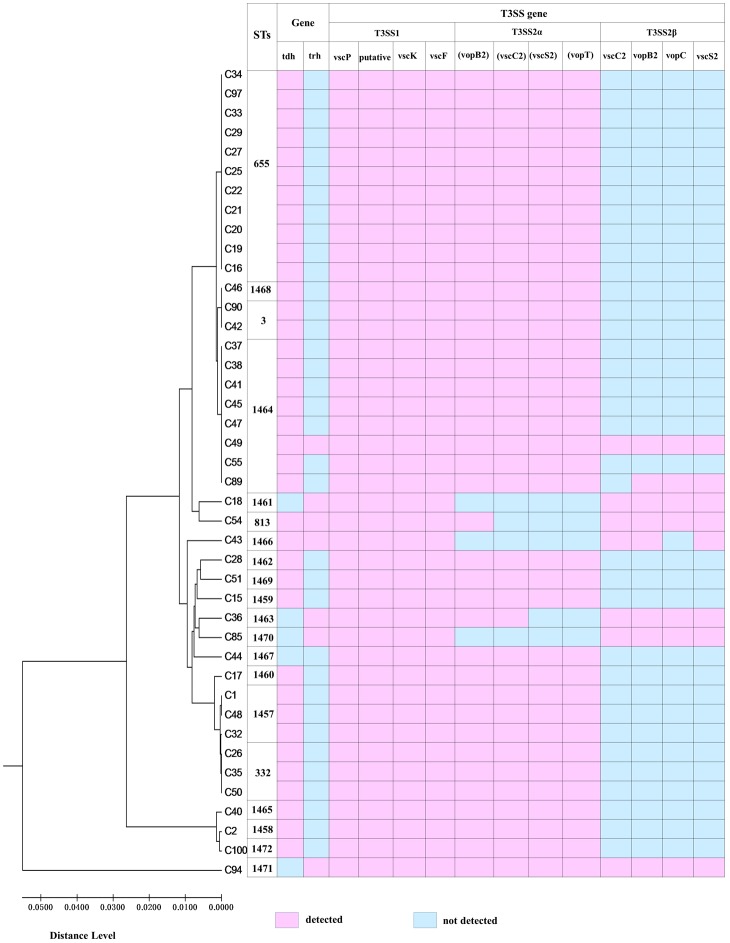
The relationship between genetic diversity and virulence-associated genes characterization of isolates. ST, sequence type.

The genetic characteristic of the *V. parahaemolyticus* isolates was analyzed by MLST. MLST classified the 42 *V. parahaemolyticus* isolates into 20 different STs (**Figure 1**), of which 16 ST were novel (ST1457, ST1458, ST1459, ST1460, ST1461, ST1462, ST1463, ST1464, ST1465, ST1466, ST1467, ST1468, ST1469, ST1470, ST1471, and ST1472). The go eBURST algorithm used in our study categorized 20 different STs into 10 singletons, one clone complexes (CC655) and Two groups (**Figure 2**). Among these, CC655 was the most prevalent clone complexes, including 22 isolates with ST655(50%), ST1464(36.4%), ST3(9.1%) and ST1468(4.5%). In addition, ST655 was the most frequent sequence type, which including 11 isolates (VPC16, VPC19 VPC20, VPC21, VPC22, VPC25, VPC27, VPC29, VPC33, VPC34, VPC97).

### Antimicrobial Resistance Profile

As shown in the **Table 3**, the isolates of *V. parahaemolyticus* were detected for different levels of antibiotic resistance. One concern, all *V. parahaemolyticus* strains were resistant to ampicillin (100%) and streptomycin (100%), followed by Cephazolin (92.9%), Kanamycin (92.9%) and Amikacin (90.5%). Isolates were also commonly resistant to Gentamicin (71.4%), Piperacillin (54.8%), and Cefoxitin (50%). In addition, all the clinical isolates showed susceptibility to six antibiotics, including Chloramphenicol, Cefotaxime, Imipenem, Ceftazidime, Trimethoprim-sulfamethoxazole and Meropenem. And only one *V. parahaemolyticus* strains (VPC2) were not found in multidrug resistance (MDR, defined as resistance to 3 or more different antimicrobials). So the multidrug resistance rate reaches to 97.6% of all 42 clinical *V. parahaemolyticus* isolates. Of these, there were 92.9% multidrug-resistant isolates showing resistance to more than five antibiotics. Strikingly, we found that four isolates showed resistance to ten antibiotics.

**Table 3:** Antimicrobial resistance profiles of 42 clinical *V. parahaemolyticus* isolates.

Classify	Antimicrobial agent	*Vibrio parahaemolyticus* (*n* = 42)		
		
		No. (%)of R	No. (%)of I	No. (%)of S
β- lactam	Ampicillin	42 (100.0)	0 (0.0)	0 (0.0)
	Amoxicillin-Clavulanic	0 (0.0)	8 (19.0)	34 (81.0)
	Piperacillin	7 (16.7)	16 (38.1)	19 (45.2)
	Cefotaxime	0 (0.0)	0 (0.0)	42 (100.0)
	Ceftazidime	0 (0.0)	0 (0.0)	42 (100.0)
	Cefoxitin	0 (0.0)	21 (50.0)	21 (50.0)
	Cephazolin	16 (38.1)	23 (54.8)	3 (7.1)
	Imipenem	0 (0.0)	0 (0.0)	42 (100.0)
	Meropenem	0 (0.0)	0 (0.0)	42 (100.0)
Tetracyclines	Tetracycline	0 (0.0)	2 (4.8)	40 (95.2)
Aminoglycosides	Amikacin	2 (4.8)	36 (85.7)	4 (9.5)
	Gentamicin	3 (7.1)	27 (64.3)	12 (28.6)
	Kanamycin	3 (7.1)	36 (85.7)	3 (7.1)
	Streptomyein	30 (71.4)	12 (28.6)	0 (0.0)
Quinolones	Ciprofloxacin	0 (0.0)	11 (26.2)	31 (73.8)
	Levofloxacin	0 (0.0)	2 (4.8)	40 (95.2)
Chloramphenicol	Chloramphenicol	0 (0.0)	0 (0.0)	42 (100.0)
Sulfonamides	Trimethoprim -sulfamethoxazole	0 (0.0)	0 (0.0)	42 (100.0)


*R, resistant; I, intermediate; S, sensitive.*

### Antimicrobial Resistance Genotypes of *V. parahaemolyticus*

The 38 antibiotic resistance genes of 6 classes of antibiotics searched in 42 pathogenic *V. parahaemolyticus* isolates are shown in Supplementary Table S3. Eight out of 38 resistance genes (*SHV*, *tet*(B), *str*A, *qnr*A, *gry*A, *qnr*B, *sul*I, *sul*II) were detected in at least one isolates. Notably, all of the clinical isolates carried two or more ARGs evaluated. Among them, *tet(*B) was the most prevalent gene, with the detection frequencies of 100%, followed by *str*A, *sul*I, *SHV*, *qnr*A, *qnr*B *gry*A, and *sul*II the detected percentage of them was 92.9, 90.5, 28.6, 28.6, 26.2,19.0, and 4.8%, respectively.

### Correlation among Virulence genes, STs, Resistance Phenotype, and Genotype

A minimum spanning tree (MST) of the sequence types (STs) that was constructed based on subtyping information, including sequence type and virulence-associated genes, is shown in **Figure 2**. As shown in **Figure 2**, We can see that the most prevalent clonal complexes were CC655, all of them were positive for virulence-related *tdh* (100%), T3SS1(100%) genes and T3SS2α (100%) genes and the majority of them were negative for the *trh* (95.5%) and T3SS2β (95.5%) genes. Notably, two distinct lineages of the T3SS2 have been described with a correlation between the presence of *tdh* with T3SS2α and *trh* with T3SS2β. From this we can observe that the virulence-related gene of *trh* and T3SS2β are co-occurrences and disappearance simultaneously. Specifically, the *tdh* (100%), T3SS1(100%) genes and T3SS2α (100%) genes were detected in all ST655 isolates.

**FIGURE 3:**
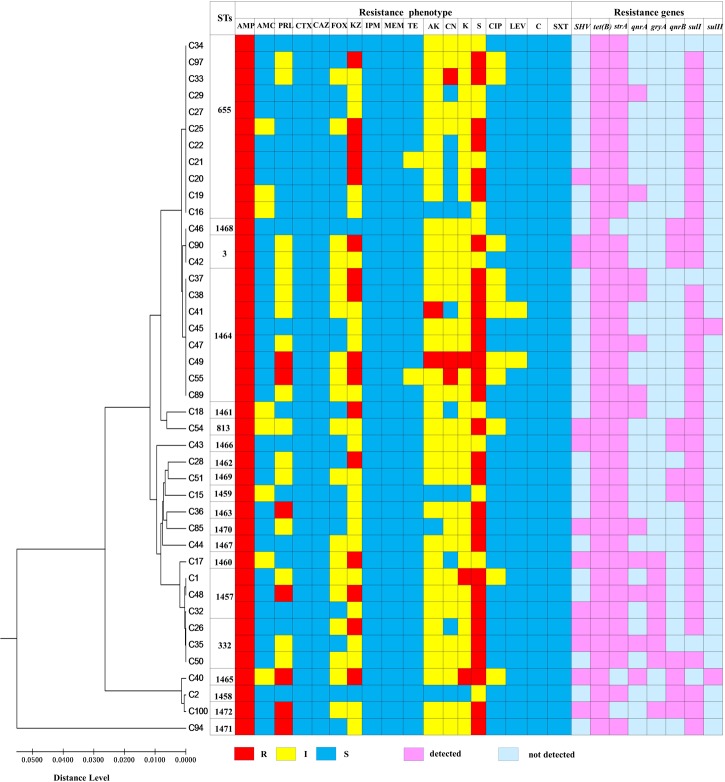
The relationship of genetic diversity, resistance phenotypic and genotypic characterization of 42 clinical *V. parahaemolyticus* isolates. ST, sequence type; R, resistant; I, intermediate; S, sensitive.

We conclude that correlation between virulence-related genes, AMR phenotypes and genotypes was absent. Likewise, as shown in **Figure 3**, genetic diversity is not apparently associated with the AMR phenotypes and genotypes.

## Discussion

In this study, we analyzed 1900 fecal specimens collected from Shanghai hospital from January, 2014 to December, 2015 and isolated 42 *V. parahaemolyticus* strains, with an isolation rate of 2.2%. Compared to other studies in China, this rate is significantly less than the 6.0% reported for southern coastal China in 2007–2012 (Li et al., 2014) and the 8.1% observed in southeastern China in 2009–2013 (Chen et al., 2016). Compared to other developing countries, our result is also less than the clinical *V. parahaemolyticus* isolation rate of 5.1% detected in Northwestern Mexico from 2004 to 2010 (Velazquez-Roman et al., 2012). That indicates the current status of *V. parahaemolyticus* infection in Shanghai is better than other regions.

The thermostable direct haemolysin (TDH), the TDH-related haemolysin (TRH) and the two type III secretion systems (T3SS1 and T3SS2) are recognized as major virulence factors in *V. parahaemolyticus* (Ceccarelli et al., 2013; Letchumanan et al., 2014; Raghunath, 2015). In our study, the hemolysin gene *tdh* was detected in 88.1% *V. parahaemolyticus* isolates, whereas the *trh* gene was present in only 7 strains. And we found that one of the clinical isolates is co-negative for *tdh* and *trh* gene. This finding is consistent with prior studies that not all clinical strains harbor these genes (Okuda et al., 1997). All *V. parahaemolyticus* strains in this study contains the T3SS1 component genes, which is also consistent with a previous study (Jones et al., 2012). The T3SS2 contains two gene clusters, T3SS2α and T3SS2β (Ritchie et al., 2012; Wang et al., 2015), which is closely related to *tdh*-positive and *trh*-positive *V. parahaemolyticus*, respectively (Jones et al., 2012). However, in this study, T3SS2α genes didn’t appear with *tdh*+ genes simultaneously, which indicates that there is high genetic heterogeneity in *V. parahaemolyticus* T3SSs.

The genetic diversity of *V. parahaemolyticus* was also investigated using MLST. Compared to other molecular methods, such as the identification of known virulence genes, phylogenetic analysis of housekeeping genes, microarray, and PFGE, MLST give a better understanding of the genetic relationships among *V. parahaemolyticus* isolates (Perez-Losada et al., 2013; Hazen et al., 2015; Ludeke et al., 2015). In this study, 42 *V. parahaemolyticus* isolates were classified into 20 sequence types (STs) with 16 novel STs. The high proportion of novel STs indicated a high genetic diversity of *V. parahaemolyticus* strains, and shows that the information content in the MLST database on this strain is still evolving. Thus, more MLST surveillances should be performed in China and the rest of world, to contributed to better understanding the genetic diversity of *V. parahaemolyticus*. Furthermore, ST655 was the most frequent hypotype in our study, which was clustered into the major clonal complex, CC655. Previous studies have been reported that ST3 belonged to the most prevalent clonal complex CC3, which is widely distributed and plays an important role in *V. parahaemolyticus* infections in multiple countries (Fuenzalida et al., 2006; Martinez-Urtaza et al., 2013; Turner et al., 2013; Haendiges et al., 2015; Han et al., 2016). There is only one locus difference between ST3 and ST655, which indicates that these STs are closely related. We recommend further research on the pandemic clonal complexes CC655 containing ST655 for management of *V. parahaemolyticus* infections.

Antibiotic treatment is necessary for controlling *V. parahaemolyticus* infections, but overuse of antibiotics has led to the generation and distribution of antimicrobial-resistant bacteria, which is becoming a major concern for human health (Ji et al., 2011; Shaw et al., 2014; Blair et al., 2015). This study also investigated the antimicrobial resistance phenotype and genotype of the 42 clinical *V. parahaemolyticus* strains. All isolates showed a high level of resistance against Ampicillin (100.0%), Streptomycin (100.0%), Cephazolin (92.9%), Kanamycin (92.8%), and Amikacin (90.5%), and eight out of 38 resistance genes (*SHV*, *tet*(B), *str*A, *qnr*A, *gry*A, *qnr*B, *sul*I, *sul*II) were detected in at least two isolates. According to this study and some previous researches (Chen et al., 2016; Xie et al., 2017), the antimicrobial resistance of clinical *V. parahaemolyticus* was significantly higher than that of environmental strains which were isolated from water (Shaw et al., 2014), aquatic products (Letchumanan et al., 2015; Lou et al., 2016; Yu et al., 2016; Xie et al., 2017) or ready-to-eat foods (Xie et al., 2016). As the human gastrointestinal tract is a conducive environment for promoting horizontal ARGs transfer (Hu et al., 2013; Theethakaew et al., 2013), we speculate that the complex gastrointestinal environment may accelerate the acquisition of antimicrobial resistance in *V. parahaemolytiucs*. The microevolution mechanisms for the different rates of acquisition of antibiotic resistance between clinical and environmental pathogens should be studied further.

## Conclusion

This study is the first comprehensive research describing the virulence, genetic diversity, antibiotic resistance phenotype, and genotype of *V. parahaemolyticus* from diarrhea patients in Shanghai. The study reveals that *tdh*, *trh* and T3SS genes are of equal importance as virulence associated factors. MLST analysis showed that the novel loci and STs points to high genetic diversity of *V. parahaemolyticus* strains isolated in Shanghai. ST655 was the most prevalent STs and this ST could have evolved from the global pandemic ST3. The antimicrobial resistance profiles indicated that the multidrug-resistant isolates were also widespread and measures to contain or slowdown the emergence of drug-resistant strains should be a top priority in China. These results add to the epidemiological data of clinical *V. parahaemolyticus* isolates in Shanghai and highlight the need for more AMR type research for managing the burden of disease caused by this pathogen in China.

## Author Contributions

HL, RT, and YL contributed equally to carrying out the experiments and writing the draft manuscript. YZ (Corresponding Author), YP, PM, and ZZ provided support for experimental design and editing of final manuscript. ZC, WC, QH assisted in completing the experiments.

## Conflict of Interest Statement

The authors declare that the research was conducted in the absence of any commercial or financial relationships that could be construed as a potential conflict of interest.

## Footnotes

1 http://pubmlst.org/v.parahaemolyticus/

2 http://www.ncbi.nlm.nih.gov/BLAST
