# Supplementary material for: A Comprehensive Epidemiological Research for Clinical Vibrio parahaemolyticus in Shanghai
Source: Front Microbiol. 2017 Jun 8;8:1043. doi: 10.3389/fmicb.2017.01043 (PMC5462930; doi:10.3389/fmicb.2017.01043)
Supplement: Supplementary file 1 [file Table_1.DOC]

**TABLE S1** Sequence of primers used in multilocus sequence typing

| Primer | Gene | Sequence of primers (5–3’) | Amplification length (bp) |
| --- | --- | --- | --- |
| *dna*E-F  *dna*E-R | *dna*E | CGRATMACCGCTTTCGCCG  GAKATGTGTGAGCTGTTTGC | 596 |
| *gyrB-*F  *gyrB-*R | *gyrB* | GAAGGBGGTATTCAAGC  GAGTCACCCTCCACWATGTA | 629 |
| *recA*-F  *recA*-F | *recA* | GAAACCATTTCAACGGGTTC  CCATTGTAGCTGTACCAAGCACCC | 773 |
| *dtdS*-F  *dtdS*-R | *dtdS* | TGG CCA TAA CGA CAT TCT GA  GAG CAC CAA CGT GTT TAG C | 497 |
| *pntA*-F  *pntA*-R | *pntA* | ACGGCTACGCAAAAGAAATG  TTGAGGCTGAGCCGATACTT | 470 |
| *pyrC*-F  *pyrC*-R | *pyrC* | AGCAACCGGTAAAATTGTCG  CAGTGTAAGAACCGGCACAA | 553 |
| *tnaA*-F  *tnaA*-R | *tnaA* | TGTACGAAATTGCCACCAAA  AATATTTTCGCCGCATCAAC | 463 |
