# Supplementary material for: A Comprehensive Epidemiological Research for Clinical Vibrio parahaemolyticus in Shanghai
Source: Front Microbiol. 2017 Jun 8;8:1043. doi: 10.3389/fmicb.2017.01043 (PMC5462930; doi:10.3389/fmicb.2017.01043)
Supplement: Supplementary file 2 [file Table_2.DOC]

**TABLE S2** Thirty-eight antimicrobial resistance genes (ARGs) and their PCR primes

| Gene | Primer | Sequence of primers (5–3’) | Amplification length (bp) | Annealing temperature (°C) | |
| --- | --- | --- | --- | --- | --- |
| β-lactam ARGs | | | | |  |
| CARB | FW | CAAGTACTTTYAAAACAATAGC | 534 | 46 | |
|  | RV | GCTGTAATACTCCKAGCAC |
| SHV | FW | GCGAAAGCCAGCTGTCGGGC | 304 | 62 | |
|  | RV | GATTGGCGGCGCTGTTATCGC |
| SHV-5 | FW | TGTTAGCCACCCTGCCGCT | 825 | 61 | |
|  | RV | GTTGCCAGTGCTCGATCAG |
| *amp*C | FW | GTGACCAGATACTGGCCACA | 822 | 61 | |
|  | RV | TTACTGTAGCGCCTCGAGGA |
| *mec*A | FW | TAATAGTTGTAGTTGTCGGGTTTG | 733 | 61 | |
|  | RV | TAACCTAATAGATGTGAAGTCGCT |
| Tetracycline ARGs | | | | |  |
| *tet*(A) | FW | GCGCTNTATGCGTTGATGCA | 387 | 62 | |
|  | RV | ACAGCCCGTCAGGAAATT |
| *tet*(B) | FW | TACGTGAATTTATTGCTTCGG | 206 | 60 | |
|  | RV | ATACAGCATCCAAAGCGCAC |
| *tet*(M) | FW | ACAGAAAGCTTATTATATAAC | 171 | 60 | |
|  | RV | TGGCGTGTCTATGATGTTCAC |
| *tet*(O) | FW | ACGGARAGTTTATTGTATACC | 171 | 60 | |
|  | RV | TGGCGTATCTATAATGTTGAC |
| *tet*(Q) | FW | AGAATCTGCTGTTTGCCAGTG | 169 | 63 | |
|  | RV | CGGAGTGTCAATGATATTGCA |
| *tet*(S) | FW | GAAAGCTTACTATACAGTAGC | 169 | 50 | |
|  | RV | AGGAGTATCTACAATATTTAC |
| *tet*(W) | FW | GAGAGCCTGCTATATGCCAGC | 168 | 64 | |
|  | RV | GGGCGTATCCACAATGTTAAC |
| *tet*(K) | FW | TCGATAGGAACAGCAGTA | 169 | 61 | |
|  | RV | CAGCAGATCCTACTCCTT |
| Aminoglycoside ARGs | | | | |  |
| aph(2′′)-Ib | FW | CTTGGACGCTGAGATATATGAGCAC | 867 | 55 | |
|  | RV | GTTTGTAGCAATTCAGAAACACCCTT |
| *str*A | FW | CTTGGTGATAACGGCAATTC | 548 | 55 | |
|  | RV | CCAATCGCAGATAGAAGGC |
| *str*B | FW | ATCGTCAAGGGATTGAAACC | 509 | 56 | |
|  | RV | GGATCGTAGAACATATTGGC |
| *aad*A | FW | ATCCTTCGGCGCGATTTTG | 283 | 56 | |
|  | RV | GCAGCGCAATGACATTCTTG |
| *aad*E | FW | ATGGAATTATTCCCACCTGA | 386 | 50 | |
|  | RV | TCAAAACCCCTATTAAAGCC |
| *aac(*6ˊ)*-*Ib | FW | TATGAGTGGCTAAATCGAT | 395 | 55 | |
|  | RV | CCCGCTTTCTCGTAGCA |
| *arm*A | FW | CCGAAATGACAGTTCCTATC | 846 | 56 | |
|  | RV | GAAAATGAGTGCCTTGGAGG |
| *rmt*B | FW | ATGAACATCAACGATGCCCT | 769 | 56 | |
|  | RV | CCTTCTGATTGGCTTATCCA |
| Quinolone ARGs | | | | |  |
| *qnr*S | FW | CCCCATGCCCGAAGTTATCA | 457 | 59 | |
|  | RV | ACTGCTTGGAGTGTGTTGGT |
| *aac(6**ˊ)-Ib-cr* | FW | ATATGCGGATCCAATGAGCAACGCAAAAACAAAGTTAG | 544 | 66 | |
|  | RV | ATAGCGAATTCTTAGGCATCACTGCGTGTTCGCTC |
| *qnr*A | FW | ATTTCTCACGCCAGGATTTG | 413 | 56 | |
|  | RV | GAGATTGGCATTGCTCCAGT |
| *gry*A | FW | CGATGTCGGTCATTGTTGGC | 455 | 61 | |
|  | RV | ATACCTACGGCGATACCGGA |
| *qnr*C | FW | TTCGATCGGACTGCTTGTGG | 438 | 59 | |
|  | RV | AACACATGGTGCAGGGGATT |
| *qnr*D | FW | GCTGGAGCTTGTCAGGGATT | 585 | 59 | |
|  | RV | TGCTGCGAGATATCATGCGT |
| *par*C | FW | GCCTAAACAACGCACGGAAA | 432 | 59 | |
|  | RV | TGACACGGGAGGTAACCAGA |
| *qnr*B | FW | TGGTGCTGTATGCACCGAAT | 453 | 58 | |
|  | RV | TCATCGCGCTGAAGAACTGT |
| Chloramphenicol ARGs | | | | |  |
| *cat*I | FW | GGTGATATGGGATAGTGTT | 349 | 60 | |
|  | RV | CCATCACATACTGCATGATG |
| *cat*II | FW | GATTGACCTGAATACCTGGAA | 567 | 60 | |
|  | RV | CCATCACATACTGCATGATG |
| *cat*III | FW | CCATACTCATCCGATATTGA | 275 | 60 | |
|  | RV | CCATCACATACTGCATGATG |
| *cat*IV | FW | CCGGTAAAGCGAAATTGTAT | 451 | 60 | |
|  | RV | CCATCACATACTGCATGATG |
| *flo*R | FW | CGCCGTCATTCCTCACCTTC | 215 | 50 | |
|  | RV | GATCACGGGCCACGCTGTGTC |
| Sulfonamide ARGs | | | | |  |
| *sul*I | FW | CGCACCGGAAACATCGCTGCAC | 163 | 63 | |
|  | RV | TGAAGTTCCGCCGCAAGGCTCG |
| *sul*II | FW | TCCGGTGGAGGCCGGTATCTGG | 191 | 63 | |
|  | RV | CGGGAATGCCATCTGCCTTGAG |
| *sul*III | FW | TCCGTTCAGCGAATTGGTGCAG | 128 | 61 | |
|  | RV | TTCGTTCACGCCTTACACCAGC |
| sulA | FW | TCTTGAGCAAGCACTCCAGCAG | 299 | 61 | |
|  | RV | TCCAGCCTTAGCAACCACATGG |
