# Supplementary material for: A Comprehensive Epidemiological Research for Clinical Vibrio parahaemolyticus in Shanghai
Source: Front Microbiol. 2017 Jun 8;8:1043. doi: 10.3389/fmicb.2017.01043 (PMC5462930; doi:10.3389/fmicb.2017.01043)
Supplement: Supplementary file 3 [file Table_3.DOC]

**TABLE S3** Antimicrobial resistance gene detection of 42 clinical *Vibrio parahaemolyticus* isolates

| Classify | Gene | | The antimicrobial resistance genotypes rate of  pathogenic *Vibrio parahaemolyticus* (%) | | | | Overall (%) |
| --- | --- | --- | --- | --- | --- | --- | --- |
|  |  | *tdh+/trh-* (n=34) | | *tdh-/trh+* (n=4) | *tdh+/trh+*  (n=3) | *tdh-/trh-*  (n=1) |  |
| β- lactam | CARB | 0(0/34) | | 0(0/4) | 0(0/3) | 0(0/1) | 0(0/42) |
|  | SHV | 26.5(9/34) | | 25.0(1/4) | 66.7(2/3) | 0(0/1) | 28.6(12/42) |
|  | SHV*-5* | 0(0/34) | | 0(0/4) | 0(0/3) | 0(0/1) | 0(0/42) |
|  | *amp*C | 0(0/34) | | 0(0/4) | 0(0/3) | 0(0/1) | 0(0/42) |
|  | *mec*A | 0(0/34) | | 0(0/4) | 0(0/3) | 0(0/1) | 0(0/42) |
| Tetracyclines | *tet*(A) | 0(0/34) | | 0(0/4) | 0(0/3) | 0(0/1) | 0(0/42) |
|  | *tet*(B) | 100(34/34) | | 100(4/4) | 100(3/3) | 100(1/1) | 100(42/42) |
|  | *tet*(M) | 0(0/34) | | 0(0/4) | 0(0/3) | 0(0/1) | 0(0/42) |
|  | *tet*(O) | 0(0/34) | | 0(0/4) | 0(0/3) | 0(0/1) | 0(0/42) |
|  | *tet*(Q) | 0(0/34) | | 0(0/4) | 0(0/3) | 0(0/1) | 0(0/42) |
|  | *tet*(S) | 0(0/34) | | 0(0/4) | 0(0/3) | 0(0/1) | 0(0/42) |
|  | *tet*(W) | 0(0/34) | | 0(0/4) | 0(0/3) | 0(0/1) | 0(0/42) |
|  | *tet*(K) | 0(0/34) | | 0(0/4) | 0(0/3) | 0(0/1) | 0(0/42) |
| Aminoglycosides | *aph*(2“)-Ib | 0(0/34) | | 0(0/4) | 0(0/3) | 0(0/1) | 0(0/42) |
|  | *str*A | 91.2(31/34) | | 100(4/4) | 100(3/3) | 100(1/1) | 92.9(39/42) |
|  | *str*B | 0(0/34) | | 0(0/4) | 0(0/3) | 0(0/1) | 0(0/42) |
|  | *aad*A | 0(0/34) | | 0(0/4) | 0(0/3) | 0(0/1) | 0(0/42) |
|  | *aad*E | 0(0/34) | | 0(0/4) | 0(0/3) | 0(0/1) | 0(0/42) |
|  | *aac(*6ˊ)*-*Ib | 0(0/34) | | 0(0/4) | 0(0/3) | 0(0/1) | 0(0/42) |
|  | *arm*A | 0(0/34) | | 0(0/4) | 0(0/3) | 0(0/1) | 0(0/42) |
|  | *rmt*B | 0(0/34) | | 0(0/4) | 0(0/3) | 0(0/1) | 0(0/42) |
| Quinolones | *qnr*S | 0(0/34) | | 0(0/4) | 0(0/3) | 0(0/1) | 0(0/42) |
|  | *aac(6ˊ)-Ib-cr* | 0(0/34) | | 0(0/4) | 0(0/3) | 0(0/1) | 0(0/42) |
|  | *qnr*A | 29.4(10/34) | | 50(2/4) | 0(0/3) | 0(0/1) | 28.6(12/42) |
|  | *gry*A | 23.5(8/34) | | 0(0/4) | 0(0/3) | 0(0/1) | 19.0(8/42) |
|  | *qnr*C | 0(0/34) | | 0(0/4) | 0(0/3) | 0(0/1) | 0(0/42) |
|  | *qnr*D | 0(0/34) | | 0(0/4) | 0(0/3) | 0(0/1) | 0(0/42) |
|  | *par*C | 0(0/34) | | 0(0/4) | 0(0/3) | 0(0/1) | 0(0/42) |
|  | *qnr*B | 26.5(9/34) | | 0(0/4) | 66.7(2/3) | 0(0/1) | 26.2(11/42) |
| Chloramphenicol | *cat*I | 0(0/34) | | 0(0/4) | 0(0/3) | 0(0/1) | 0(0/42) |
|  | *cat*II | 0(0/34) | | 0(0/4) | 0(0/3) | 0(0/1) | 0(0/42) |
|  | *cat*III | 0(0/34) | | 0(0/4) | 0(0/3) | 0(0/1) | 0(0/42) |
|  | *cat*IV | 0(0/34) | | 0(0/4) | 0(0/3) | 0(0/1) | 0(0/42) |
|  | *flo*R | 0(0/34) | | 0(0/4) | 0(0/3) | 0(0/1) | 0(0/42) |
| Sulfonamides | *sul*I | 88.2(30/34) | | 100(4/4) | 100(3/3) | 100(1/1) | 90.5(38/42) |
|  | *sul*II | 5.9(2/34) | | 0(0/4) | 0(0/3) | 0(0/1) | 4.8(2/42) |
|  | *sul*III | 0(0/34) | | 0(0/4) | 0(0/3) | 0(0/1) | 0(0/42) |
|  | *sul*A | 0(0/34) | | 0(0/4) | 0(0/3) | 0(0/1) | 0(0/42) |
| Overall (%) |  | 8.0(104/1292) | | 11.0(15/136) | 11.4(13/114) | 7.9(3/38) | 10.3(165/1596) |
